# Efficacy and safety of the serratus anterior plane block (SAP block) for pain management in patients with multiple rib fractures in the emergency department: a retrospective study

**DOI:** 10.1007/s00068-024-02597-6

**Published:** 2024-07-17

**Authors:** Sossio Serra, Domenico Pietro Santonastaso, Giuseppe Romano, Alessandro Riccardi, Stefano Geniere Nigra, Emanuele Russo, Mario Angelini, Vanni Agnoletti, Mario Guarino, Claudia Sara Cimmino, Michele Domenico Spampinato, Raffella Francesconi, Fabio De Iaco

**Affiliations:** 1 Emergency Department, Maurizio Bufalini Hospital, Cesena, 47521 Italy; 2 Anestesia and Intensive Care Unit, Emergency Department, Maurizio Bufalini Hospital, Cesena, 47521 Italy; 3 SC Pronto Soccorso e Medicina d’Urgenza, Ospedale Santa Corona, Pietra Ligure, Savona, 17027 Italy; 4 UOC MEU Ospedale CTO-AORN dei Colli Napoli, Napoli, 80131 Italy; 5 Department of Translational Medicine and for Romagna, University of Ferrara, Via A. Moro 8, Ferrara, 44124 Italy; 6 Struttura Complessa di Medicina di Emergenza Urgenza Ospedale Maria Vittoria, ASL Città di Torino, Torino, 10144 Italy

**Keywords:** Pain management, Trauma, Chest trauma, SAP block, Stopthepain, Emergency medicine

## Abstract

**Purpose:**

Chest trauma is a severe and frequent cause of admission to the emergency department (ED). The serratus anterior plane (SAP) block seems to be an effective method of pain management; however, data on efficacy and safety of a single SAP block performed in the ED by emergency physicians (EP) are limited. This study aimed to compare SAP block performed by the EP in the ED plus standard therapy to standard therapy alone in terms of pain severity at 0-3-6-12-18 and 24 h, total opioid consumption (milligrams of morphine equivalents, MME), respiratory function (SpO2/FiO2 ratio), and adverse events (i.e. pneumothorax, infections in the site of injection, or Local Anaesthetic Systemic Toxicity syndrome due to SAP block) in the first 24 h.

**Methods:**

This retrospective, monocentric study included adult patients admitted to the Sub-intensive Care Unit (SICU) of the ED with multiple rib fractures between 01/2022 and 03/2023.

**Results:**

156 patients (65.4% male; median age 62 years; median injury severity score 16; median thoracic trauma severity score 8) were included. 75 (48.2%) underwent SAP block. Patients undergoing SAP block showed significantly less pain 3–6–18 h after a single block, required less MME (0 [0–20] vs. 20 [0–40], *p* < 0.001), showed higher SpO2/FiO2 ratio, and no adverse events were reported.

**Conclusion:**

The SAP block, in combination with standard therapy, appeared to be more effective in providing pain relief than standard therapy alone in patients admitted to the SICU for traumatic rib fractures.

## Introduction

Blunt chest trauma is a serious presentation to the emergency department (ED) that can lead to acute impairment of respiratory function, including respiratory failure and death, due to acute damage to the thoracic structures or worsening of pre-existing pathological conditions [1]. Multiple rib fractures play a significant role in blunt chest trauma and are associated with a high risk of morbidity and mortality, particularly in elderly patients [2, 3]. In the early stages, pneumothorax, haemothorax, and lung contusions are the most frequently observed injuries in chest trauma [2], which can lead to acute respiratory failure and respiratory arrest. In the late stages, uncontrolled severe pain leads to (i) reduced thoracic excursion with reduced functional residual respiratory capacity [4] and (ii) impaired cough with reduced secretion clearance [5, 6], secondary atelectasis, and a higher risk of infection. Secondary pneumonia is one of the principal risk factors for in-hospital death due to subsequent hypoxemia and an inflammatory systemic response affecting multiple organ functions [7], possibly leading to admission to the intensive care unit and death [8]. Therefore, effective pain management should be considered a fundamental step in the treatment of chest trauma, which should be achieved as soon as possible [7, 9] to promote pulmonary ventilation and good respiratory rehabilitation [10–13] and to improve the patient’s overall outcome [3, 5, 11, 14].

Various analgesic methods and combinations have been evaluated for rib fracture-related pain treatment, including paracetamol and non-steroidal anti-inflammatory drugs, systemic opioids, patient-controlled analgesia, lidocaine patches, and regional analgesia techniques such as thoracic epidural and paravertebral catheters, and intercostal and paravertebral blocks [5, 15, 16]. However, the use of traditional regional analgesia techniques *may be* limited because of underlying injuries (e.g., spinal injuries), contraindications (e.g., trauma-related coagulopathy and anticoagulant medications), multimorbidity (especially in elderly trauma patients), and inability to position the patient during the acute phase [17]. In the last decade, interest in chest wall fascial blocks, such as the serratus anterior plane block (SAP block), erector spine plane block, and rhomboid intercostal block, has increased [4, 15]. The SAP block [18–20] is a fascial block traditionally used for surgical anaesthesia and postoperative analgesia in thoracic procedures as an alternative to more invasive paravertebral and epidural blocks. It involves injecting a long-acting local anaesthetic, such as ropivacaine, bupivacaine, or levobupivacaine, into a virtual anatomical space between two fasciae, one surrounding the latissimus dorsi muscle and the other covering the serratus anterior muscle. The injection of the local anaesthetic in this virtual space, distant from both the pleural cavity and the major nerve or vascular branches, and the use of ultrasound for real-time guidance of the technique significantly reduce the likelihood of complications such as thoracic injury (pneumothorax), neurovascular damage, and systemic complications in comparison to other techniques [21, 22].

Injecting the local anaesthetic into this space at the level of the 5th intercostal space on the midaxillary line (or between the 3rd and 7th ribs) allows the anaesthetic to reach the nerve fibres of the cutaneous lateral branch of the intercostal nerves of the hemithorax [23]. It is particularly effective in controlling pain in the anterior and lateral chest areas between T2 and T9 [18], while its efficacy in managing pain in more posterior regions is questionable [24]. The local anaesthetic is also capable of blocking nerve conduction of the branches innervating the ribs, rib-sternal joints, and cartilage-rib muscles and is potentially extremely helpful in controlling pain following thoracic trauma [23].

Despite increasing evidence regarding the efficacy and safety of fascial nerve blocks, this novel technique is largely underutilised. A report from the U.S. National Trauma Database indicates that only 3 per cent of patients with thoracic trauma eligible for fascial nerve blocks have access to this treatment [25].

The objective of this study was to investigate the efficacy and safety of SAP block performed by emergency physicians in the ED and sub-intensive care unit (SICU) compared with standard care alone in terms of pain control, opioid consumption, and adverse events in the first 24 h after admission in adult patients with multiple rib fractures.

## Methods

This is a retrospective observational study based on electronic medical records of adult patients admitted to the SICU of the ED of the “M. Bufalini” Hospital in Cesena, Italy, from January 2022 to March 2023, with multiple rib fractures due to traumatic injuries, regardless of the presence of other traumatic injuries. Patients (i) admitted to other hospital care units, (ii) dismissed at home, (iii) lost to follow-up or with a lack of data in the first 24 h, or (iv) with contraindications to opioids were excluded from the study.

The “M. Bufalini” hospital in Cesena is a level I trauma centre designated as an integrated system for caring for trauma patients of the Emilia Romagna Regional Health Service in northern Italy and treats approximately 65,000 patients annually. The ED of the “M. Bufalini” Hospital in Cesena is equipped with four physicians 24 h a day, seven days a week, and provides unscheduled care for patients whose condition requires immediate treatment, including patients with hemodynamic instability and multiple traumas. The ED has seven monitored beds available for prolonged observation (up to 24 h) and a 24-bed Sub-Intensive Care Unit (SICU), eight of which are monitored and provide the ability to perform invasive and non-invasive ventilation, invasive and non-invasive blood pressure monitoring, and treatment of patients with clinical and hemodynamic instability. The SICU is managed by an emergency physician who works alternately in the ED and the SICU.

From June 2022, the emergency physicians working in the ED and the SICU of the “M. Bufalini” Hospital received a theoretical course and practical training for the ultrasound-guided (USG) SAP block procedure, as the SAP block procedure was introduced in emergency care for the treatment of blunt chest trauma.

**Fig. 1:**
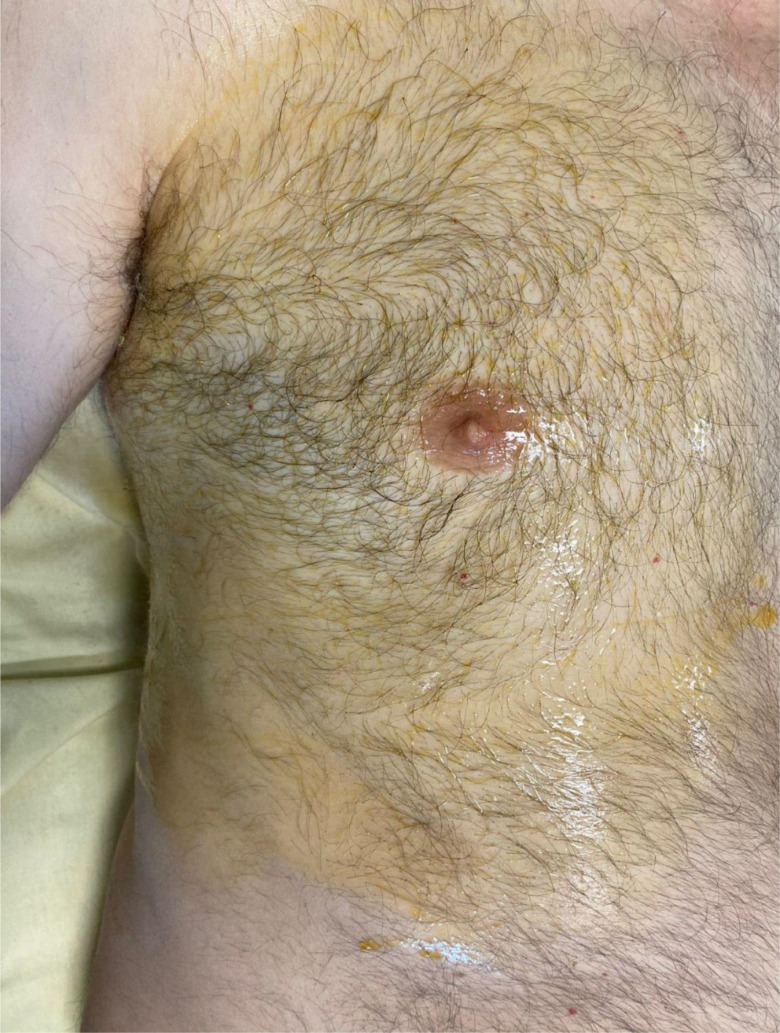
Patient in the supine position with the right hemithorax undergoing disinfection and field delineation

**Fig. 2:**
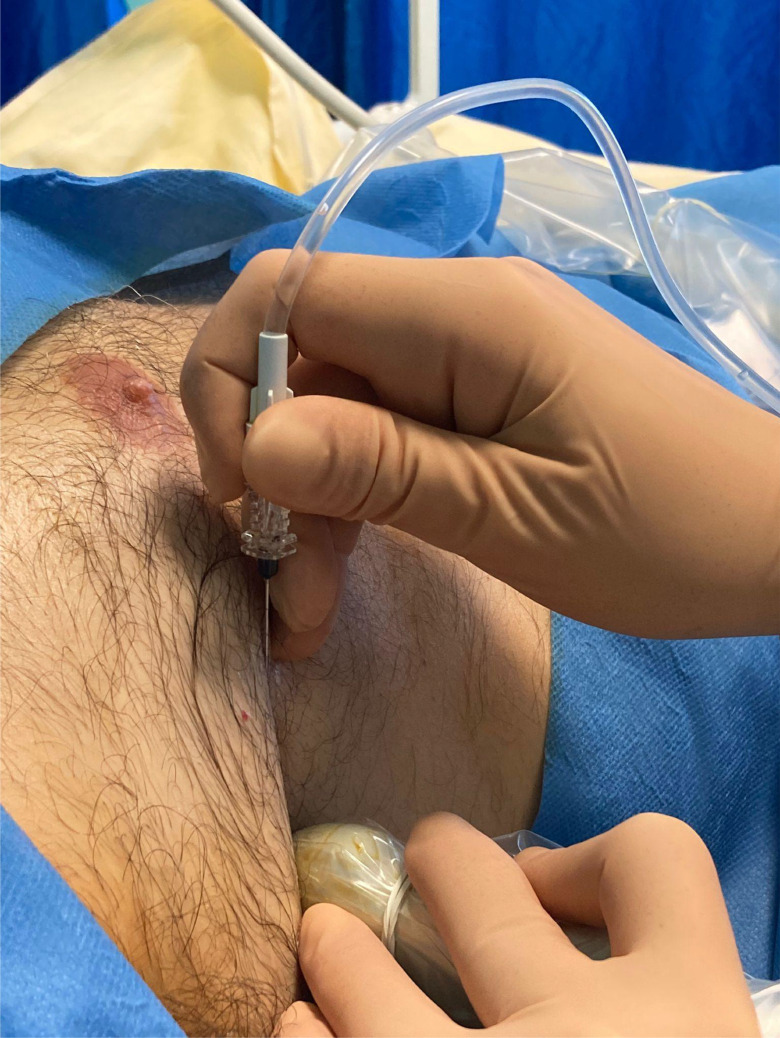
Using a sterile technique, the probe is placed in the mid-axillary anterior axilla at the height of the fifth intercostal space. With the non-dominant hand, the linear ultrasound probe is held, while with the dominant hand, the needle is orientated to perform the block

**Fig. 3:**
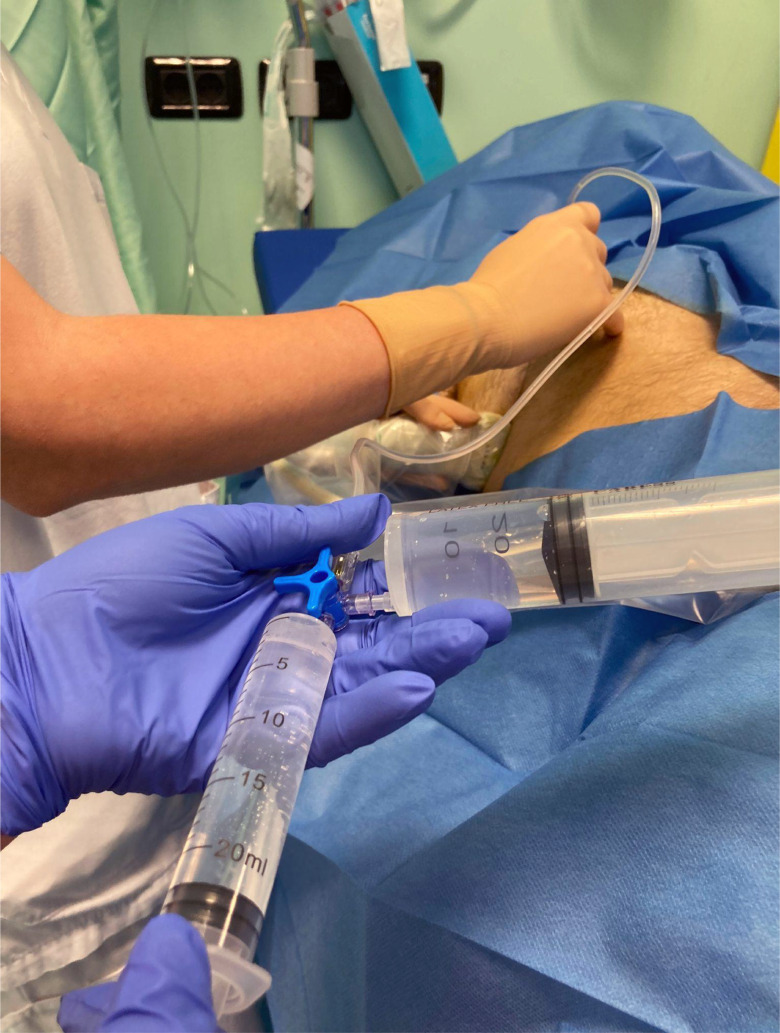
The technique is performed by two operators: the first operator controls the ultrasound probe and the position of the needle within the fascial planes via ultrasound guidance, and the second operator alternately injects diluted saline or ropivacaine according to the first operator’s instructions. The saline is injected to confirm the correct position of the needle in the fascial space; once the proper location of the needle is confirmed, the injection of ropivacaine is performed

**Fig. 4:**
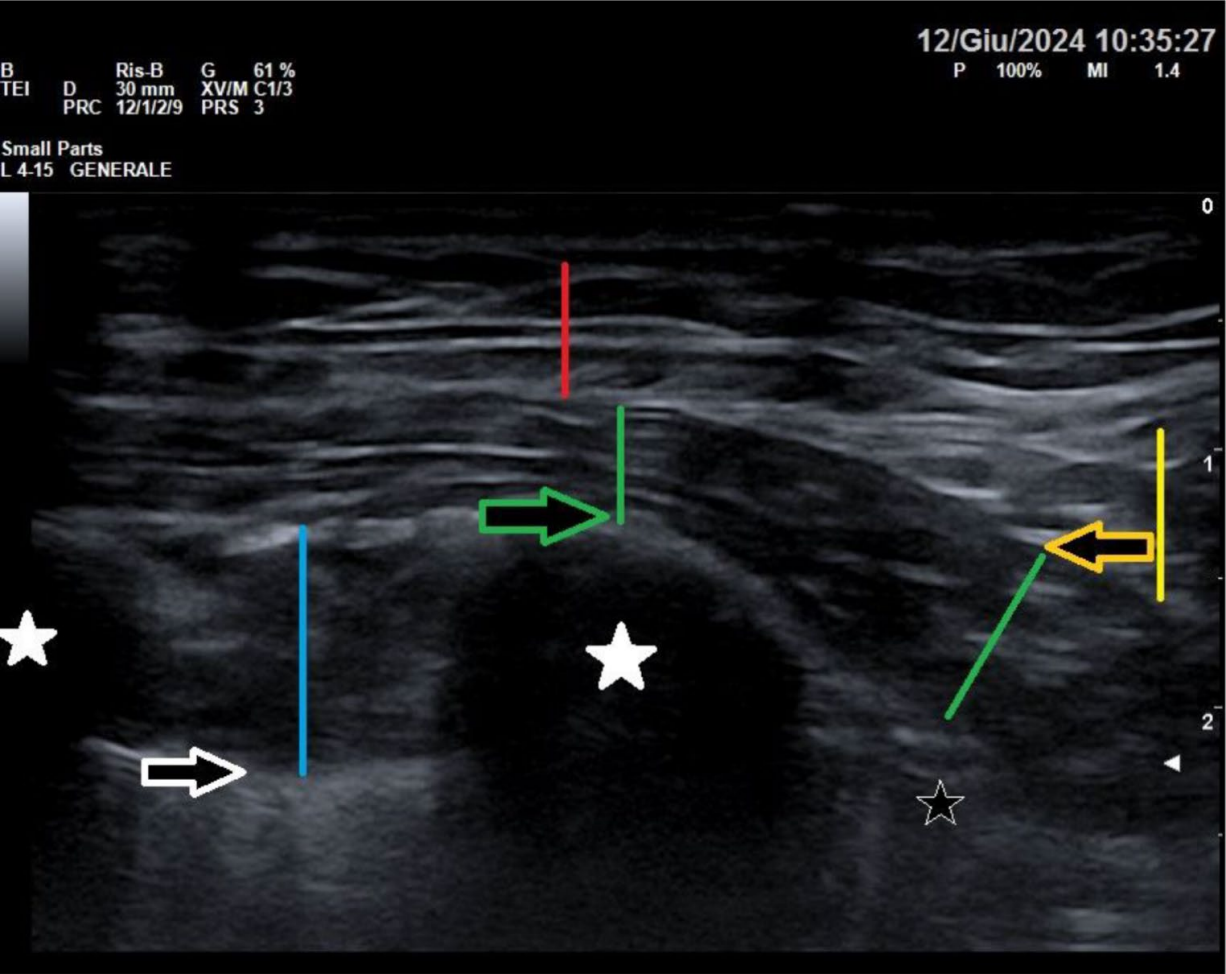
Image obtained with a high-frequency linear probe positioned as in Fig. 2: The red line indicates the thickness of the skin and subcutis; the yellow line indicates the thickness of the latissimus dorsi muscle; the green lines identify the serratus anterior muscle, which lies on the ribs; the blue line identifies the intercostal muscles; the white stars identify the ribs; the yellow arrow identifies the fascial plane between the latissimus dorsi and the serratus anterior, site of the superficial SAP block; the green arrow identifies the fascial plane between the serratus anterior muscle and the rib, site of the deep SAP block; the black star identifies presence of emphysema which prevents visualisation of deeper structures

**Fig. 5:**
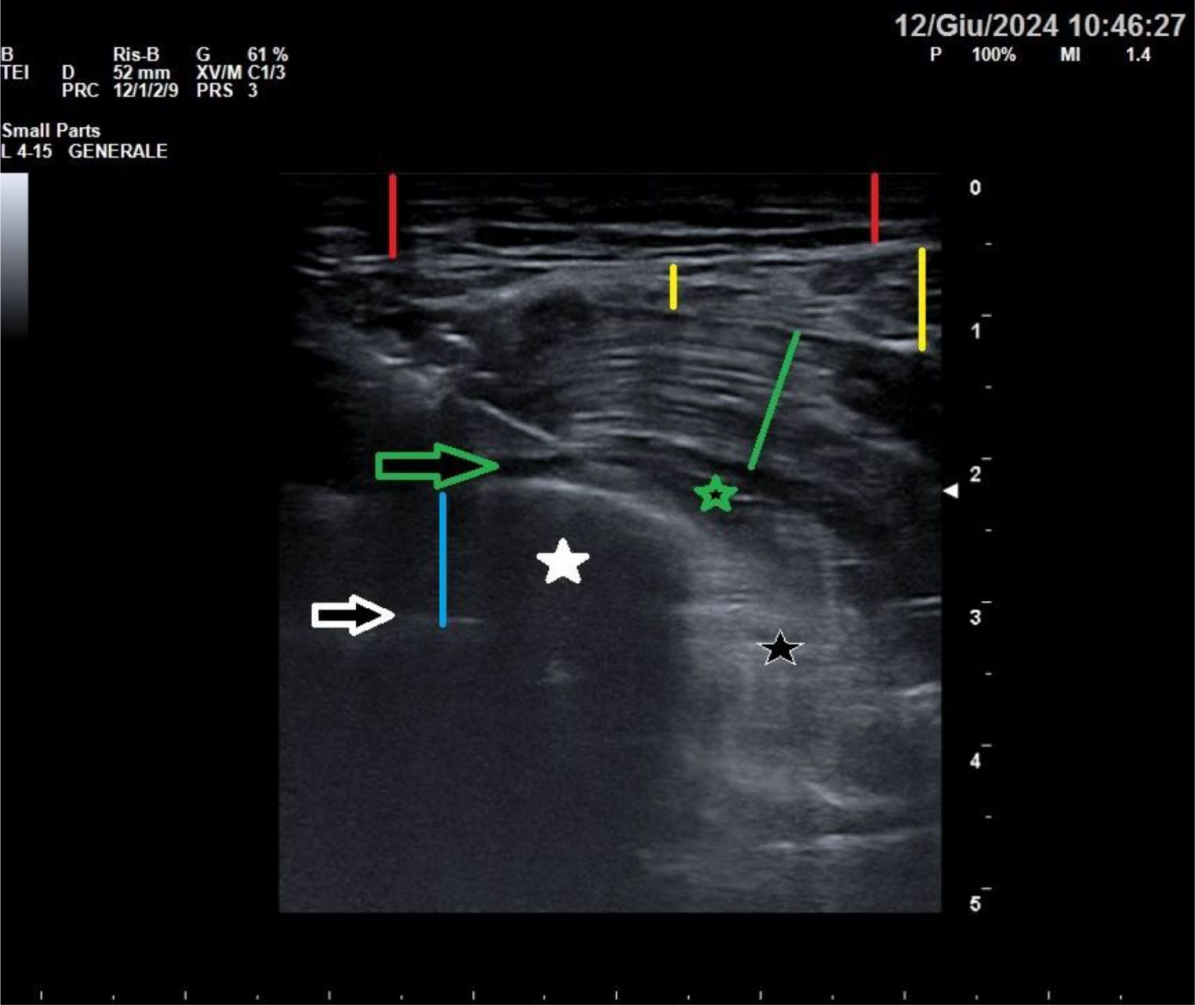
illustrates the deep SAP block, in which the local anaesthetic is injected into the fascia below the serratus anterior muscle and the ribs. The red lines indicate the thickness of the skin and subcutis; the yellow lines indicate the thickness of the latissimus dorsi muscle; the green line identifies the serratus anterior muscle; the blue line identifies the intercostal muscles; the white star identifies the rib; the green arrow identifies the fascial plane between the serratus anterior muscle and the rib, site of the deep SAP block; the green star indicates the hydrodissection of the fascia below the serratus anterior, showing the spreading of the local anaesthetic between the serratus anterior and the rib; the black star identifies presence of emphysema which prevents visualisation of deeper structures

The SAP block course was organised as follows: each emergency physician took part in an 8-hour theoretical course and 4-hour practical training at the bedside, conducted by hospital anaesthetists experienced in the SAP block technique. USG-SAP block procedures were performed at the bedside in the ED or immediately after hospitalisation in the SICU. The SAP block was proposed to patients in the following cases: (i) patients aged > 18 years with chest trauma and severe pain, expressed as an NRS > 6; (ii) spontaneous breathing; (iii) more than two rib fractures; (iv) no haemodynamic instability; and (v) Glasgow Coma Scale > 13. Contraindications to SAP block were considered: (i) allergy to amide-type local anaesthetics, (ii) history of peripheral neuropathy, (iii) severe hepatic or renal insufficiency and (iv) infection at the injection site. During the SAP block, the patients were placed in supine or lateral positions. They underwent standard multiparametric monitoring, including non-invasive blood pressure, a three-lead electrocardiogram, and peripheral pulse oximetry. Sterile USG was performed using a portable ultrasound system (SonoSite M-Turbo; SonoSite Inc., WA, USA). A linear high-frequency probe (6–13 MHz) was placed in the midaxillary line to identify and scan the target ribs, latissimus dorsi, and serratus anterior muscles in the sagittal plane. A 50 mm long 22-gauge needle (Echoplex+, Vygon, Ecouen-France) was inserted into the fascial plane, and 70–100 mg of 7.5% ropivacaine (1-1.5 mg/kg) plus 0.9% saline up to 30 ml of total volume of was injected under direct vision. The linear spread of the local anaesthetic between the serratus anterior muscle and ribs (in case of deep SAP block) or between latissimus dorsi and serratus anterior muscles (in case of superficial SAP block) was directly observed to confirm the procedure. For bilateral rib fractures, SAP block was performed only on the most injured side. See Figs. 1, 2, 3, 4 and 5 for a detailed explanation of the technique.

All patients were treated with analgesic therapy according to the World Health Organisation analgesic ladder for nociceptive pain: mild pain (level 1), non-opioids (paracetamol, non-steroidal anti-inflammatory drugs [NSAIDs]) ± adjuvants; moderate pain (level 2), weak opioids (tramadol) ± non-opioids ± adjuvants; and severe pain (level 3), strong opioids (morphine, fentanyl) ± non-opioids ± adjuvants [26]. Patients with pain due to rib fracture(s) who received analgesic therapy in combination with an SAP block were included in the SAP block group. In contrast, patients who received only analgesic treatment were included in the standard therapy alone group.

The primary endpoint of this study was the NRS pain score at 3, 6, 12, 18, and 24 h in the SAP block group compared to the standard therapy alone group. The secondary endpoints were (i) total opioid consumption in the first 24 h, (ii) pulmonary function expressed by SpO2/FiO2 ratio, and (iii) occurrence of adverse events in the USG SAPB plus standard therapy group compared to the standard therapy group alone.

Pain and opioid therapy were monitored and recorded in patients in the control group during the first 24 hours after admission to the ED and in patients in the SAP block group 24 hours after the SAP block was performed. Each patient’s 24-hour opioid consumption was converted to equivalent doses of intravenous morphine using an opioid conversion Table [27]. Pain was measured using an 11-point numerical rating scale (NRS), with 0 representing ‘no pain’ and 10 representing ‘worst pain ever”. Comorbidities were recorded using the Charlson Comorbidity Index (CCI) [28], trauma severity using the Injury Severity Score (ISS) [29], and Thoracic Trauma Severity Score (TTSS) [30]. Adverse events evaluated were: occurrence of pneumothorax due to SAP block; infections in the site of injection of the local anaesthetic, and occurrence of any sign or symptom compatible with the local Anaesthetic Systemic Toxicity (LAST) syndrome, including metallic taste, tremor, restlessness or drowsiness, seizures, cardiac arrhythmias, or cardiac arrest. The vital signs recorded were respiratory rate (RR), peripheral oxygen saturation (SpO2), SpO2/FiO2 ratio [31–33], systolic blood pressure (SBP), diastolic blood pressure (DBP), and heart rate (HR) on admission and after 3, 6, 12, 18, and 24 hours. The decision to initiate oxygen therapy, high-flow nasal cannula oxygen therapy (HFNC), continuous positive airway pressure (CPAP) oxygen therapy, noninvasive ventilation (NIV), or orotracheal intubation was based on individual clinical judgment.

Continuous data are expressed as median and interquartile range; differences between two independent groups were compared using the Mann-Whitney U test, while the Wilcoxon test was used to compare two paired groups. Categorical data are expressed as absolute numbers and percentages; differences between two independent groups were compared using the Pearson chi-square test. Statistical analyses were performed using IBM SPSS version 25 (IBM Corp.) and MedCalc version 17.6 (MedCalc Software).

This study was conducted following the current Helsinki Declaration and approved by the local ethics committee, the “CET-CEROM—Comitato etico della Romagna”, dated December 13, 2023, approval code: 7379/2023. The STROBE checklist was used to prepare this manuscript [34].

## Results

**Fig. 6:**
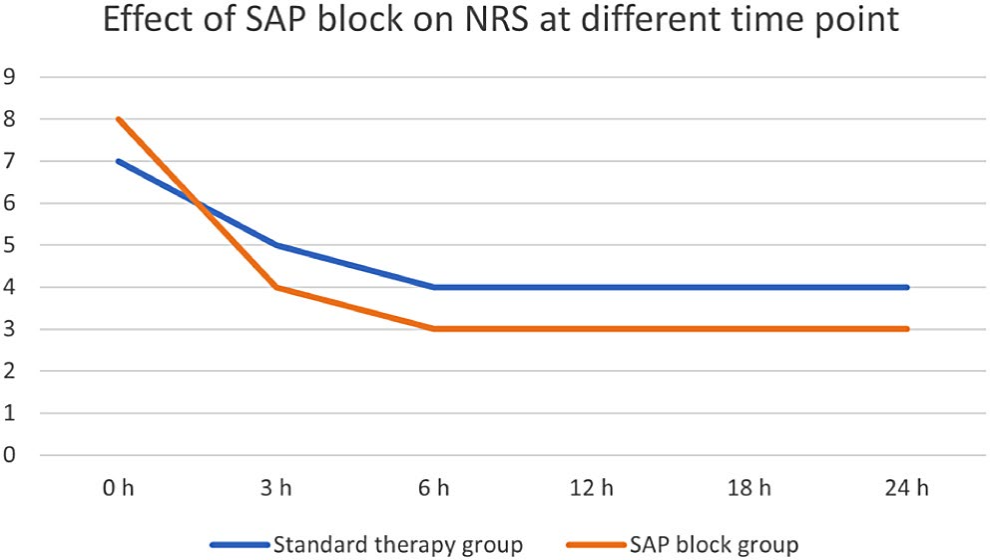
NRS at different time points in the SAP block group vs. the standard therapy group

During the study period, 191 patients with chest trauma were admitted to the ED. However, 35 patients were excluded because they were admitted to the intensive care unit (ICU), 8 were sent home from the ED, 5 were admitted to the internal medicine department, and 3 were lost to follow-up (two patients were transferred to the surgical care unit, one after 6 h from admission and the other after 12 h, one patient was transferred to another hospital, and none of them underwent SAP block). Finally, 156 patients were included in the study, 102 of whom were male (65.4%), with a median age of 62 years (IQR 52–74) and a median CCI of 3 (IQR 1–5). In addition, 32 patients had bilateral rib fractures (20.5%), with a median of 6 fractured ribs (IQR 4–7), 4 (IQR 0–5) on the right side, and 4 (IQR 0–6) on the left side, with a median TTSS of 8 (IQR 6–9) and a median ISS of 16 (IQR 9–18). A total of 75 patients (48.2%) underwent USG SAP block in the ED. Patients who underwent SAP block had lower TTSS (7 [IQR 6–8] vs. 8 [7–10], *p* = 0.026) and higher NRS on admission (8 [8–9] vs. 7 [5–8], *p* < 0.001) than those who did not undergo the procedure. There were no differences in sex, median age, median CCI, ISS, vital signs, or the number of fractured ribs. Two patients developed severe respiratory failure during their stay in the SICU and underwent orotracheal intubation (1 in the SAP block group and 1 in the standard therapy group); two patients developed pneumonia (1 in the SAP block group and 1 in the standard therapy group); and no patient underwent IHM. Patients who underwent SAP block received a median dose of 80 mg ropivacaine (IQR 80–100), and none of the patients experienced a specific complication due to the administration of the local anaesthetic. Regarding the primary endpoint, patients who underwent SAP block had significantly less pain in the first 24 h than patients who underwent standard therapy only. As reported in Table 1; Fig. 6, at each time point considered, NRS was significantly lower in the SAP group than in the standard therapy-only group, except for NRS at 12 (*p* = 0.059) and 24 h (*p* = 0.458). Regarding the secondary endpoint, patients who underwent SAP block received significantly lower opioid doses (0 [0–20] MME vs. 20 [0–40] MME, *p* < 0.001). (see Fig. 6).

According to the respiratory function, patients who did not undergo SAP block showed a significant decrease in respiratory function during the first 24 h with an SpO2/FiO2 ratio of 392 [IQR 335–457] at admission vs. 325 [271–452] at 24 h, p 0.001, while those who underwent SAP block showed a stable SpO2/FiO2 ratio (412 [IQR 355–457] at admission vs. 457 [331–461] at 24 h, p 0.326), showing a higher SpO2/FiO2 ratio at each subsequent time point evaluated and requiring less frequent use of HFNC (3 [4] vs. 32 [39.5], *p* < 0.001). Evaluating only patients with bilateral rib fractures, the SAP block group showed a significant reduction in pain perception after 6 h (NRS = 4 [IQR 1–4], vs. NRS = 4 [IQR 3–5], *p* = 0.034) and after 12 h (median NRS = 4 [IQR 2–4], vs. median NRS = 4 [IQR 2–5], *p* = 0.045), whereby less MME was administered (median MME = 10 [IQR 0–20] vs. 20 [IQR 0–40], *p* < 0.001). For the general characteristics of the population included, see Table 1.

## Discussion

The present study suggests that in adult traumatic patients with a median TTSS of 8 and a median ISS of 16 admitted to the SICU of the ED, USG SAP block, associated with standard therapy, is more effective than standard therapy alone in reducing pain during the first 24 h after block execution and at each time point, except at 12 and 24 h after enrolment. Moreover, SAP block was associated with a significantly lower dose of opioids administered and significantly better respiratory function, without differences in terms of adverse events compared to standard therapy alone. Although the 2011 Montreal Declaration [35] recognises the identification and treatment of pain as a fundamental human right, inadequate analgesia is a significant problem in the emergency department. Pain is the leading cause of admission to the ED, being associated with the main reason for admission in 61% of cases and the main reason for admission in up to 52.2% of cases [36]. Nevertheless, it is often underestimated and undertreated. In 1989, the term “oligoanalgesia” was introduced, which accurately describes how pain is frequently not adequately treated [37, 38]. As shown in the present study, a high proportion of admitted patients experience moderate to severe pain. In patients with rib fractures, the presence of pain severely restricts thoracic excursion and the ability to excrete secretions, leading to alveolar collapse and pulmonary atelectasis and increasing the risk of bacterial over-infection and patient death.

Traditional analgesics, such as paracetamol, NSAIDs, and opioids, are used to treat pain. However, paracetamol is only effective to a limited extent and is mainly used for mild pain. NSAIDs have numerous contraindications and adverse effects that are more common in elderly patients (renal insufficiency, concomitant treatment with antiplatelet agents or anticoagulants, and previous gastrointestinal bleeding). In contrast, opioids can lead to sedation, especially in frail patients. The use of low-dose ketamine (LDK) for analgesia is widespread [39], and there is also evidence for its use in trauma [40]. The SAP block has been successfully used to treat acute pain in multiple rib fractures [41]. In the current study, patients in the SAP block group achieved faster pain control than those in the standard therapy group (Fig. 1). They showed significantly better pain control at all time intervals except the 12- and 24-hour marks.

Although patients who underwent SAP block reported higher pain scores on admission, the patients in the SAP block group had significantly less pain just 3 h after admission. Achieving optimal pain control is essential for providing high-quality care to patients. The 2020 European Society of Emergency Medicine guidelines on managing acute pain in the emergency department recommend that no more than 25 min should be allowed for pain relief (if appropriate) [42]. Despite this, in the ED, pain management takes an average of 90 min [43], only 14–32% of patients receive adequate pain relief [41], and in 41% of patients, pain remains unchanged during the ED stay [43].

The SAP block is effective in less than 60 min [44]. However, recent studies have shown that the SAP block can reduce perceived pain by an average of 5 points (IQR ± 4) after 30 min [45], from 8/10 to 0/10 within 30 min [46], to 2/10 after 15 min [47], and a mean reduction in pain score of 6.8 within 10 min. The positive effect lasts up to 30 h and is contextually significant in improving respiratory function [48].

The SAP block provides analgesia while avoiding the systemic effects of other drugs, with an exclusively local effect [19, 49], greater efficacy than the traditional pharmacological approach [50], and a significant reduction in opioid consumption [51, 52]. It is worth noting that although performing a bilateral SAP block may be the better choice for patients with fractures of both ribs, patients with bilateral rib fractures also experienced better pain management and a reduced amount of medication for pain control despite a unilateral SAP block.

In addition, the SAP block has also been reported to be effective in allowing early and safe discharge of patients in whom hospitalisation would have been indicated only for pain control [53] and in reducing chronic pain after rib fractures [54]. Multimodal analgesic therapy based on both traditional approaches and local anaesthesia ensures the best outcome, especially in multiple rib fractures with secondary respiratory dysfunction [9, 55, 56], by significantly improving diaphragmatic excursion [57] and global pulmonary ventilatory function [45]. In the present study, patients who underwent SAP block showed a significantly higher SpO2/FiO2 ratio at each time point than those who did not undergo SAP block, in whom the SpO2/FiO2 ratio decreased significantly, requiring HFNC in a higher percentage of cases. As illustrated in Table 1, patients who did not undergo SAP block had a substantially higher proportion of pneumothorax and a higher TTSS, which can significantly affect the SpO2/FiO2 ratio and the need for respiratory support. However, no differences in the rates of pneumonia, orotracheal intubation, or in-hospital mortality were found between patients receiving SAP block and standard therapy and those receiving standard therapy alone. The effect of SAP block on pulmonary function needs further studies to be fully established. Moreover, the present study was not designed to show differences in the rates of these adverse events, and the lack of differences is likely due to the small number of patients included.

In addition, the use of fascial blocks in the ED may be particularly beneficial in different populations, including the elderly, where it has been shown to reduce the incidence of delirium in hospitals by up to 35 per cent [58], herpes zoster-related pain [59], burns [60], and thoracostomy [61].

The SAP block relies on detecting anatomical structures that are easily recognisable in ultrasound, as they are superficial structures, and these characteristics make the SAP block a straightforward fascial block to perform [62]. Two different approaches to performing the SAP block have been described: the superficial and deep approaches [18]. In the superficial approach, the local anaesthetic is injected into the virtual space between the serratus anterior and latissimus dorsi muscles. In contrast, in the deep approach, the local anaesthetic is injected into the space between the rib and serratus anterior. Theoretically, the superficial SAP block can be advantageous because the long thoracic nerve innervating the serratus anterior muscle is on its surface. Therefore, superficial SAP blocks can potentially block this nerve completely while also blocking the anterior branches of the intercostal nerves, as can deep SAP blocks. In contrast, deep SAP blocks cannot block the long thoracic nerve [63]. The superficial SAP block appears to have better analgesic efficacy in terms of pain scores, opioid consumption, time to first analgesic rescue and dermatomal spread in patients who have undergone mastectomy with axillary clearance [64] and in patients undergoing thoracoscopic surgery [63]. However, the clinical advantages of one approach over another are still controversial. Edwards et al. [65] and Piracha et al. [66] have demonstrated the superior efficacy of the deep SAP block. This is probably because it is easier to accurately locate the virtual space when the needle tip is placed above the ribs than the challenge of identifying the interfascial space between the latissimus dorsi and the serratus anterior to administer the local anaesthetic correctly.

Furthermore, the efficacy and safety of a fascial block depends on the specific type and dosage of the long-acting local anaesthetic used and the volume administered. The choice of local anaesthetic depends on factors such as the onset of action, duration of action, and the extent of motor blockade required. Rosenblatt et al. recommended 20 ml of 0.25% bupivacaine or 0.2% ropivacaine [67]. However, in the present study, the SAP blocks were performed with 30 ml of 0.23–0.33% ropivacaine. Ropivacaine is a longer-acting local anaesthetic that is less toxic than bupivacaine [68–70]. Although the recommended concentration is 0.2%, 0.33% allows for a faster onset of action [71].

Biswas et al. [72] showed that injecting a larger total volume of local anaesthesia leads to a broader and more consistent spread of anaesthesia, irrespective of the area injected. However, the clinical significance of these differences should be better clarified. In their randomised controlled trial, Kunigo et al. [73] demonstrated that the time until patients required the first analgesic rescue dose was comparable between patients receiving a total volume of 40 ml and those receiving a total volume of 20 ml, although a volume of 40 ml produced a greater area of sensory loss in the craniocaudal direction.

While this study highlights the efficacy of 30 ml of 0.23–0.33% ropivacaine for deep SAP block, further research is required to determine the effectiveness of using different volumes of long-acting local anaesthetic and the role of superficial or deep SAP blocks in the treatment of pain following rib fractures.

The SAP block is a relatively safe procedure. A recent meta-analysis by Jack et al. reported that SAP blocks did not result in adverse events compared to the control groups. In particular, no pneumothorax, nerve damage, hemorrhagic complications, infections, or Local Anaesthetic Systemic Toxicity (LAST) syndromes were noted, even in cases where they were performed in patients on anticoagulant therapy [74]. However, most of the studies included in the meta-analysis by Jack et al. investigated the role of the SAP block during thoracotomy or video-assisted thoracoscopic surgery, and the evidence for thoracic trauma included only case series and reports. The present retrospective study suggests that SAP block can be introduced into clinical practice after only a single theoretical and practical course. It appears to be a safe manoeuvre even in ED patients with multiple thoracic rib fractures [43, 60, 75]. Although the study was not designed to demonstrate the safety of SAP block due to the rare occurrence of local anaesthetics system toxicity (LAST) syndrome [71, 76], no adverse events were reported that could be associated with the administration of ropivacaine at the fascial site.

Recently, the SABRE randomised clinical trial confirmed the efficacy and safety of SAP block performed in the ED in terms of pain control, opioid reduction, and SAP block-related adverse events. Despite the trial reporting no data on the effect of SAP block on respiratory function, no significant differences were noted regarding secondary pneumonia, length of stay, or 30-day mortality. However, the trial was not conceived to detect differences in these important outcomes, and subsequent studies are needed to explore these crucial aspects [77].

This study has several limitations, including a non-randomised design in a single trauma centre, recruitment limited by exclusion criteria, and the availability of an emergency physician able to perform the SAP block. Moreover, formal sample size was not calculated, and the lack of differences between the two groups may be related to the small number of patients included. The time study was limited to the first 24 h. It included patients regardless of the thoracic region in which the fractures were localised (including unilateral and bilateral rib fractures) and irrespective of the presence of other injured areas. Despite these data showing an improvement in pain perception with less need for MME, the results may differ in cases of bilateral USG-SAP blocks or patients with only thoracic injuries, especially regarding MME required to achieve adequate pain management and overall complications. The control group had a higher TTSS, and the SAP block group had significantly higher pain scores (NRS) at baseline. Still, despite this data, patients treated with SAP block significantly improved their pain scores.

Moreover, the administration of prehospital analgesia was not reported, which may have influenced the NRS reported at admission and 3 h after inclusion and the total dose of opioids administered in both groups. The present study is also limited to controlling for the placebo effect of the SAP block. In addition to the inclusion and exclusion criteria, several other factors may have influenced the selection of patients who underwent SAP block. These factors could include patient preferences and the emergency physician’s experience level, potentially introducing selection bias. Additional randomised prospective studies are necessary to establish definitive conclusions regarding the efficacy and safety of SAP block. This study reflects the experience of a single centre, in which patients were treated by different emergency physicians after a single course. Despite the safety and relative ease with which the manoeuvres can be performed, continuous training is essential for maintaining and improving skills. Subsequently, various studies are required to determine the best training system and ability to perform the SAP block over time.

## Conclusions

Serratus anterior plane block, or SAP block, performed by emergency physicians in the emergency department in conjunction with standard therapy in patients with severe pain due to traumatic rib fractures, appeared to be more effective than standard therapy alone in relieving pain at 3, 6, and 18 h after a single block, with a significantly lower amount of opioid required to achieve adequate analgesia, allowing for better respiratory function, and no adverse events reported.

In conclusion, integrating the SAP block into multimodal analgesic therapy in the emergency department could be a safe and efficient approach for pain management in patients with multiple rib fractures.

**Table 1:** General characteristics of the included patients

		All patients, *N* = 156	Standard therapy group, *N* = 81 (51.9%)	SAP block group, *N* = 75 (48.2)	*p* value
Sex Male		102 (65.4)	49 (60.5)	53 (70.7)	0.85
Age, years		62 (52–74)	61 (51–74)	64 (55–74)	0.758
ISS		16 (9–18)	16 (9–19)	13 (9–16)	0.097
CCI		3 (1–5)	3 (1–5)	3 (2–5)	0.481
TTSS		8 (6–9)	8 (7–10)	7 (6–8)	0.026
Total fractured ribs		6 (4–7)	5 (4–7)	6 (5–7)	0.31
Number of fractured ribs on the right		4 (0–5)	4 (0–5)	4 (0–6)	0.701
Number of fractured ribs on the left		4 (0–6)	3 (0–6)	4.5 (0–6)	0.234
Presence of pneumothorax (mono or bilateral)		50 (32)	35 (43.2)	15 (20)	0.002
Presence of haemothorax (mono or bilateral)		9 (5.7)	5 (6.2)	4 (5.3)	0.822
Chest tube positioned (mono or bilateral)		5 (3.2)	3 (3.7)	2 (2.7)	0.785
Presence of lung contusion(s) (mono or bilateral)		42 (26.9)	19 (23.5)	23 (30.7)	0.310
Presence of any head and neck injury		27 (17.3)	16 (19.8)	11 (14.7)	0.401
Presence of any face injury		9 (16.1)	3 (3.7)	6 (8)	0.542
Presence of any abdomen injury		16 (10.3)	9 (11.1)	7 (9.3)	0.715
Presence of any extremity injury (including pelvis)		14 (8.9)	7 (8.6)	7 (9.3)	0.880
Presence of any external injury		51 (32.7)	27 (33.3)	24 (32)	0.859
SBP, mmHg		140 (125–150)	139 (125–145)	140 (130–150)	0.883
DBP, mmHg		80 (70–80)	80 (70–80)	80 (70–87)	0.02
HR, ppm		80 (70–85)	80 (70–80)	80 (70–89)	0.11
SpO2, %		97 (95–98)	97 (95–98)	96 (95–98)	0.249
Fio2, %		23 (21–28)	25 (21–29)	22 (21–28)	0.173
SpO2/FiO2 at admission		401 (342–457)	392 (335–457)	412 (355–457)	0.326
RR, bpm		20 (18–20)	20 (18–21)	18 (16–20)	0.25
RR at 3 h, bpm		20 (18–22)	20 (18–22)	20 (18–22)	0.478
SpO2/FiO2 at 3 h		407 (320–457)	344 (280–457)	452 (331–461)	0.005
RR at 6 h, bpm		20 (18–20)	20 (18–20)	20 (18–20)	0.605
SpO2/FiO2 at 6 h		384 (316–457)	327 (268–457)	457 (327–461)	< 0.001
RR at 12 h, bpm		20 (18–20)	20 (18–20)	19 (18–20)	0.192
SpO2/FiO2 at 12 h		415 (274–457)	326 (262–452)	452 (334–466)	0.001
RR at 18 h, bpm		19 (18–20)	20 (18–20)	18 (18–20)	0.126
SpO2/FiO2 at 18 h		388 (316–457)	327 (257–452)	457 (327–466)	0.001
RR at 24 h, bpm		19 (18–20)	20 (18–20)	18 (18–20)	0.361
SpO2/FiO2 at 24 h		442 (316–457)	325 (271–452)	457 (331–461)	< 0.001
Ropivacaine, mg		80 (80–100)	-	80 (80–100)	-
NRS at admission		8 (6–9)	7 (5–8)	8 (8–9)	< 0.001
NRS at 3 h		4 (3–5)	5 (4–6)	4 (2–5)	0.012
NRS at 6 h		4 (2–5)	4 (2–6)	3 (2–4)	< 0.001
NRS at 12 h		3 (2–4)	4 (3–5)	3 (2–4)	0.059
NRS at 18 h		3 (2–4)	4 (3–5)	3 (2–4)	< 0.001
NRS at 24 h		3 (2–5)	4 (2–5)	3 (2–4)	0.458
Paracetamol g/die	0	1 (0.6)	1 (1.2)	0 (0)	0.04
	2	2 (1.3)	1 (1.2)	1 (1.3)	
	3	103 (66)	64 (79)	40 (53.3)	
	4	50 (32)	15 (18.5)	34 (45.3)	
MME, mg		20 (0–20)	20 (0–40)	0 (0–20)	< 0.001
NSAIDS		2 (1.3)	2 (2.5)	0 (0)	0.171
LDK		1 (0.6)	1 (1.2)	0 (0)	0.334
Oxygen therapy via nasal cannula or facial mask		30 (19.2)	12 (14.8)	18 (24)	0.146
HFNC		35 (22.4)	32 (39.5)	3 (4)	< 0.001
CPAP		0 (0)	0 (0)	0 (0)	-
NIV		0 (0)	0 (0)	0 (0)	-
Orotracheal intubation		2 (1.3)	1 (1.3)	1 (1.3)	0.956
Pneumonia		2 (1.3)	1 (1.2)	1 (1.3)	0.956
Complication due to SAP block		0 (0)	0 (0)	0 (0)	-
Length of Stay, days		4.5 (3–6)	5 (4–6)	4 (3–6)	0.342
IHM		0 (0)	0 (0)	0 (0)	-

*Note* bpp: breaths per minute; CCI: charlson comorbidity index; CPAP: continuous positive airway pressure; DBP: diastolic blood pressure; HFNC: high-flow nasal cannula; HR: heart rate; IHM: in-hospital mortality; ISS: injury severity score; LDK: low-dose ketamine; MME: mg of morphine equivalents; NIV: non-invasive ventilation; NRS: numeric rating scale (0 no pain-10 maximum pain ever); NSAIDs: non-steroidal anti-inflammatory drugs; ppm: pulses per minute; RR: respiratory rate; SAP: serratus anterior plane; SBP: systolic blood pressure; TTSS: thoracic trauma severity score. All data are expressed as number (N) and percentage (%) or median and interquartile range

## Data Availability

No datasets were generated or analysed during the current study.
